# Study on the Differences between Traditional Chinese Medicine Syndromes in NYHA I–IV Classification of Chronic Heart Failure

**DOI:** 10.1155/2019/2543413

**Published:** 2019-02-03

**Authors:** Jun Shi, Liangtao Luo, Jing Chen, Juan Wang, Huihui Zhao, Wei Wang

**Affiliations:** ^1^Beijing University of Chinese Medicine (Institute of Traditional Chinese Medicine; Beijing Key Laboratory of TCM Syndrome and Formula; Ministry of Education Key Laboratory of TCM Syndrome and Formula), Bei San Huan Dong Lu, Chao Yang District, Beijing 100029, China; ^2^Basic Medical School of Inner Mongolia Medical University, Hohhot 010010, China

## Abstract

**Objectives:**

This study investigated the distribution of characteristics of traditional Chinese medicine syndromes and their association with symptoms in 1027 patients with chronic heart failure (CHF).

**Methods:**

An observational study was performed by researchers, collecting data from 1036 patients with CHF from 24 Chinese medicine hospitals from May 2009 to December 2014. Due to incomplete information from nine patients, 1027 patients with CHF were analysed. The distribution of syndromes in CHF and association between high-frequency syndromes and symptoms were investigated.

**Results:**

The primary syndromes were qi deficiency, blood stasis, fluid retention, yin deficiency, phlegm turbidity, and yang deficiency. The primary sites of disease were the heart, kidney, lung, and spleen. In patients with CHF of differing cardiac function, there was no significant difference in the frequency of yin deficiency (P>0.05). The distribution of yang deficiency was significantly different between New York Heat Association (NYHA) classes II, III, and IV and between classes I+II and III+IV (P<0.05). The frequency of phlegm turbidity was significantly different between NYHA classes II and III, between classes III and IV, and between classes I+II and III+IV (P<0.05). The frequency of fluid retention was significantly different between NYHA classes I and IV, between classes II, III, and IV, and between classes I+II and III+IV (P<0.05). Regarding associations between syndromes and symptoms, qi deficiency was diagnosed in 87.43% of patients with insomnia and spiritlessness; blood stasis in 84.85% of patients with spontaneous sweating + cyanosis of the lips; fluid retention in 75% of patients with a hard pulse and oedema; and yin deficiency in 72.92% of patients with feverish sensation in the chest, palms, and soles and spontaneous sweating.

**Conclusions:**

The frequency of yang deficiency and fluid retention was higher and that of phlegm turbidity was lower in classes III and IV than in classes I and II.

## 1. Introduction

Chronic heart failure (CHF), which is the name given to symptomatic chronic cardiac insufficiency, mainly refers to abnormal cardiac function caused by various reasons that obstruct the heart's pumping, causing insufficient blood output and finally leading to the insufficient perfusion of organs and tissues, accompanied by stasis of the systemic and/or pulmonary circulation. It is the final stage of heart disease of various causes and the main cause of death; thus, it has a high morbidity and mortality clinically [1, 2]. Although substantial progress has been made in the diagnosis and treatment of heart failure, its incidence is still increasing [3]. According to data from the Centers for Disease Control [4], approximately 5.7 million adults in the United States suffer from heart failure, and approximately half of these patients die within five years of their initial diagnosis. In fact, deaths from heart failure account for a large fraction of all deaths from chronic diseases. There are currently 4 million patients with heart failure in China, as the incidence of CHF increases year by year. The Chinese Guidelines for the Diagnosis and Treatment of Heart Failure published in 2014 by the Chinese Society of Cardiology clearly establish that heart failure has a high incidence and is one of the most important contemporary cardiovascular diseases [5].

In recent years, traditional Chinese medicine (TCM) played an increasingly prominent role in the prevention and treatment of CHF as it focuses on integral syndrome differentiation and adjustment and has the special advantage in promoting the functional recovery of various organs, reducing adverse reactions, and improving the quality of life of patients [6–10]. Treatment based on syndrome differentiation is the advantage and essence of TCM. Syndromes are the basis for the diagnosis and treatment of diseases in TCM. The syndrome is the essential summary of the aetiology, pathogenesis, and pathological state of a certain stage in the course of disease, which manifests itself as clinical symptoms and signs that can be observed. Traditional Chinese medicine holds that heart failure is mainly caused by heart disease or other diseases involving the heart, resulting in a deficiency of qi, blood, yin, and yang, or that the heart is invaded by six exogenous pathogenic factors, thereby damaging the heart. The syndrome is dynamic throughout the occurrence, development, and prognosis of CHF. Therefore, studies on the distribution and evolution of the TCM syndromes of CHF can help understand the pathogenetic changes, development, and prognosis of CHF and improve clinical syndrome differentiation. It is of great significance for the early identification of and intervention in high-risk patients, further reducing risk of future cardiovascular events. Based on previous research [11], this study clinically investigated the distribution of syndrome characteristics and the association of high-frequency syndromes with symptoms in patients with CHF caused by coronary heart disease from 24 Chinese medicine hospitals, in order to provide guidelines for syndrome differentiation and treatment of CHF.

## 2. Materials and Methods

### 2.1. General Methods

A total of 1036 patients with CHF treated in 24 Chinese medicine hospitals (including two hospitals of integrated traditional Chinese and Western medicine, China-Japan Friendship Hospital and Chengdu Integrated Traditional Chinese and Western Medicine Hospital) from May 2009 to December 2014 were enrolled in the study. The data used to support the findings of this study have not been made available to protect the subjects' privacy. There were 590 (56.95%) male and 446 (43.05%) female patients. The male-to-female ratio was 1.32:1. The average age was 67.54 ± 10.40 years. However, four patients had unknown cardiac function and five had no description of their syndrome. Therefore, 1027 patients with CHF were analysed.

### 2.2. Criteria

#### 2.2.1. Diagnostic Criteria

*(1) Diagnostic Criteria of CHF*. CHF was diagnosed according to the Guidelines for the Diagnosis and Treatment of Chronic Heart Failure (2007) in China [12]. Cardiac function was classified according to the New York Heart Association (NYHA) classification (1928).

*(2) Diagnostic Criteria of TCM*. Qi deficiency, yang deficiency, and yin deficiency were diagnosed according to the deficiency syndrome differentiation criteria established by the Professional Committee of Deficiency Syndrome and Geriatrics, Chinese Association of Integrative Medicine, in 1986 [13]. Blood stasis was diagnosed according to the diagnostic criteria for blood stasis developed by the Professional Committee of Promoting Blood Circulation to Remove Blood Stasis, Chinese Association of Integrative Medicine, in 1986 [14]. Qi stagnation, phlegm turbidity, water retention, cold coagulation, heat syndrome, and dampness syndrome were diagnosed according to the National Standard of the People's Republic of China - Clinical Diagnosis and Treatment Terms in Chinese Medicine: Syndrome [15].

*(3) Standards for Symptoms*. The symptoms were recorded according to the Standard Terminology for Common Clinical Symptoms in Chinese Medicine [16].

#### 2.2.2. Inclusion Criteria

Inclusion criteria included basic heart disease history; signs and symptoms of CHF; a diagnosis of CHF confirmed using echocardiography; a diagnosis of coronary heart disease confirmed using coronary angiography, coronary artery computed tomography, a previous history of acute myocardial infarction, pathological Q waves on electrocardiogram (ECG), or electrocardiographic stress test and myocardial radionuclide imaging; no history of hypertension or blood pressure <160/100 mmHg on medication; age >18 years and ≤80 years; and provided informed consent.

#### 2.2.3. Exclusion Criteria

Exclusion criteria included acute myocardial infarction, acute myocarditis, cardiogenic shock, or severe arrhythmia with hemodynamic changes; current concomitant infection, as evidenced by any one of fever, white blood cell count >10 × 10^9^/L and neutrophil > 85%, or patchy shadows on chest radiographs; severe hepatic insufficiency (parameters of liver function >2 times higher than normal); Ccr >20%, Scr >3 mg/dl or >265 *μ*mol/L; haematological primary disease; malignancy; pregnancy or lactation; mental disease; and infectious disease.

### 2.3. Measures

A clinical epidemiological questionnaire regarding CHF was developed based on previous research [6] and three rounds of expert consultation. The questionnaire was completed by three appropriately trained and qualified researchers for each enrolled patient within 24 hours of admission. All completed questionnaires were sent to the person in charge of the research group at Beijing University of Chinese Medicine. Completion of the questionnaire was monitored by special clinical monitors.

### 2.4. Statistical Methods

Responses were double entered into an EpiData 3.1 database by two separate individuals. Database consistency was checked. Inconsistencies were corrected according to the data source. Statistical analysis with two-sided tests was performed using SPSS 23.0 software. P≤0.05 was considered significant.

The relationship between high-frequency syndromes and symptoms was analysed using the Apriori algorithm in SPSS Modeler 14.0. The association rules [17] try to discover correlations between different data items regarding the same event, that is, to identify all the subsets of items and attributes frequently occurring in the transaction, and the correlations between the items. In TCM research, they are mainly used to analyse the correlation between different drugs, between symptoms and drugs, between syndrome and symptoms, and between pathogenesis and symptoms [18]. In this study, association rules were mainly used to explore the relationship between high-frequency syndromes and symptoms. Support [19] is defined as the proportion of transactions containing both A and B of the total number of transactions. If the proportion of transactions containing A is expressed as P(A), then support = P(A&B). Confidence [19] is defined as the proportion of transactions containing both A and B to those containing A and denoted as P(A&B)/P(A).

## 3. Results

### 3.1. Cardiac Function Classification in CHF

As categorised using the NYHA cardiac function classification, 11 patients (1.06%) were class I, 289 (27.9%) were class II, 611 (58.98%) were class III, 120 (11.58%) were class IV, and five (0.48%) were unknown.

### 3.2. Distribution of Syndromes

Of the 1036 patients, five patients had unknown cardiac function and four had no description of their syndrome. Therefore, 1027 patients with CHF were analysed. The syndromes identified in the 1027 patients, in descending order of frequency, were qi deficiency, blood stasis, fluid retention, yin deficiency, phlegm turbidity, yang deficiency, blood deficiency, qi stagnation, others, cold coagulation, and dryness-heat; the disease sites were heart, kidney, lung, spleen, liver, stomach, and gallbladder. The results are shown in Tables 1 and 2.

### 3.3. Distribution of Syndromes in Patients with CHF in Different Cardiac Function Classes

In the 11 patients with class I CHF, in descending order of frequency, blood stasis, qi deficiency, yin deficiency, phlegm turbidity, fluid retention, and yang deficiency syndromes were seen; the disease sites were the heart, kidney, spleen, lung, and liver. In the 286 patients with class II CHF, in descending order of frequency, qi deficiency, blood stasis, phlegm turbidity, yin deficiency, fluid retention, yang deficiency, blood deficiency, qi stagnation, cold coagulation, and dryness-heat syndromes were seen; the disease sites were the heart, spleen, kidney, lung, liver, stomach, and gallbladder. In the 610 patients with class III CHF, in descending order of frequency, qi deficiency, blood stasis, fluid retention, yang deficiency, yin deficiency, phlegm turbidity, blood deficiency, and qi stagnation syndromes were seen; the disease sites were the heart, lung, kidney, spleen, liver, stomach, and gallbladder. In the 120 patients with class IV CHF, in descending order of frequency, qi deficiency, blood stasis, fluid retention, yang deficiency, phlegm turbidity, yin deficiency, and qi stagnation were seen; the disease sites were the heart, kidney, spleen, lung, stomach, liver, and gallbladder. The results are shown in Tables 3 and 4.

### 3.4. Comparison of Distribution of Yin Deficiency, Yang Deficiency, Phlegm Turbidity, and Fluid Retention between Different Cardiac Function Classes

The differences in the distribution of syndromes in patients with CHF between different cardiac function classes were mainly seen in four syndromes: yin deficiency, yang deficiency, phlegm turbidity, and fluid retention. Therefore, the distribution of the four syndromes in different cardiac function classes was compared using a row-by-column chi-square test. In patients with CHF of different cardiac function classes, there was no significant difference in the distribution of yin deficiency (P > 0.05); however, there was a significant difference in the distribution of yang deficiency, phlegm turbidity, and fluid retention (P < 0.05).

The distribution of yang deficiency was significantly different between NYHA classes II, III, and IV, and between classes I + II and III + IV (P<0.05). The distribution of phlegm turbidity was significantly different between NYHA classes II and III, between classes III and IV, and between classes I + II and III + IV (P < 0.05). The distribution of fluid retention was significantly different between NYHA classes I and IV; between classes II, III, and IV; and between classes I + II and III + IV (P < 0.05). The results are shown in Table 5.

### 3.5. Analysis of Association Rules between High-Frequency Syndromes and Symptoms in Patients with CHF

**Figure 1:**
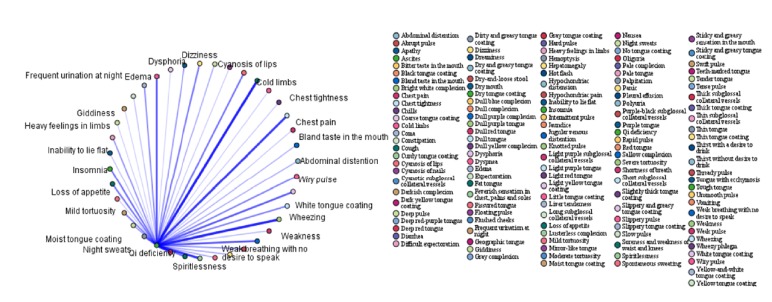
Association rules between qi deficiency and symptoms.

**Figure 2:**
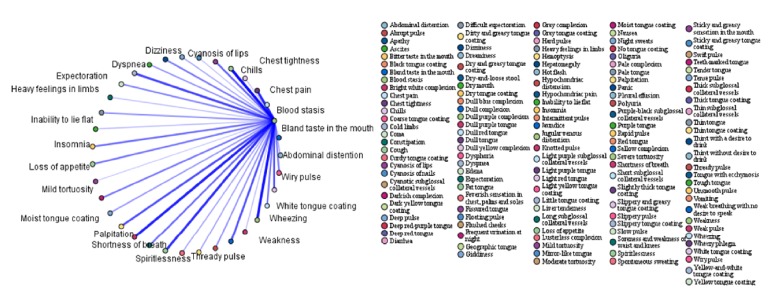
Association rules between blood stasis and symptoms.

**Figure 3:**
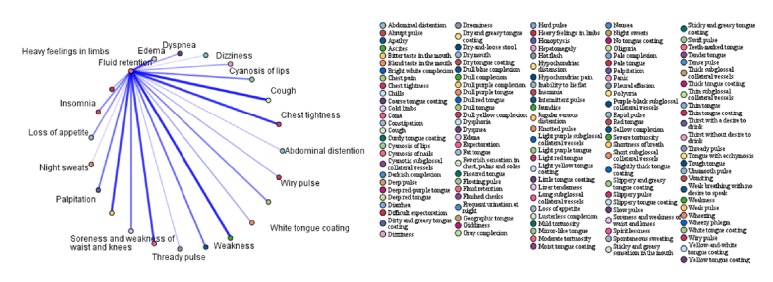
Association rules between fluid retention and symptoms.

**Figure 4:**
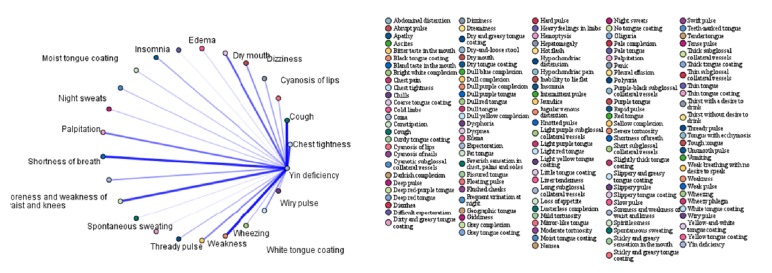
Association rules between yin deficiency and symptoms.

In order to further investigate the association between syndromes and symptoms, the association rules between the four highest-frequency syndromes (qi deficiency, blood stasis, fluid retention, and yin deficiency) and symptoms were analysed. The top 15 association rules ranked in order of confidence value are shown in Tables 6–9. The association rules between high-frequency syndromes and symptoms are presented in Figures 1–4. In the association analysis graphs, the thickness of the line represents the degree of association. The thicker the line is, the higher the degree of association is, and vice versa. The results are detailed in the following sections.

#### 3.5.1. Association Rules between qi Deficiency and Symptoms

In patients with CHF and qi deficiency, the common symptoms were insomnia + spiritlessness, thready pulse + shortness of breath, moist tongue coating + spiritlessness, insomnia + shortness of breath, soreness and waist and knee weakness + spiritlessness, insomnia + weak breathing with no desire to speak, insomnia + weakness, thready pulse + weakness, wiry pulse + spiritlessness, and insomnia + palpitation according to the association rules between qi deficiency and symptoms. Among them, the highest confidence was obtained for qi deficiency => insomnia + spiritlessness (87.43%). In other words, qi deficiency was diagnosed in 87.43% of cases where insomnia + spiritlessness were reported. The results are shown in Table 6 and Figure 1.

#### 3.5.2. Association Rules between Blood Stasis and Symptoms

According to the association rules between blood stasis and symptoms, the common symptoms were spontaneous sweating + cyanosis of the lips, night sweats + cyanosis of lips, thready pulse + cyanosis of the lips, oedema + cyanosis of lips, wiry pulse + cyanosis of the lips, chest pain + weakness, chest pain + cyanosis of the nails + spiritlessness, abdominal distension + cyanosis of the lips, and cyanosis of the nails + chest tightness. Among them, the highest confidence was obtained for spontaneous sweating + cyanosis of the lips (84.85%). In other words, blood stasis was diagnosed in 84.85% of cases where spontaneous sweating + cyanosis of the lips were reported. The results are shown in Table 7 and Figure 2.

#### 3.5.3. Association Rules between Fluid Retention and Symptoms

According to the association rules between fluid retention and symptoms, the common symptoms were hard pulse + oedema, difficult expectoration + oedema, hypochondriac distension + spontaneous sweating, nausea + hypochondriac distension, hypochondriac distension + inability to lie flat, hypochondriac distension + oedema, thirst without desire to drink + thready pulse, oliguria + thready pulse, nausea + oedema, and thirst without desire to drink + insomnia. Among them, the highest confidence was obtained for hard pulse + oedema (75%). In other words, fluid retention was diagnosed in 75% of cases where hard pulse + oedema were reported. The results are shown in Table 8 and Figure 3.

#### 3.5.4. Association Rules between Yin Deficiency and Symptoms

According to the association rules between yin deficiency and symptoms, the common symptoms were feverish sensation in the chest, palms, and soles + spontaneous sweating, red tongue + thready pulse, red tongue + dry mouth, feverish sensation in the chest, palms, and soles + night sweats, feverish sensation in the chest, palms, and soles + dysphoria, fissured tongue + dry mouth, tongue red + wiry pulse, feverish sensation in the chest, palms, and soles + dizziness, feverish sensation in the chest, palms, and soles + thready pulse, feverish sensation in the chest, palms, and soles + dry mouth, and feverish sensation in the chest, palms, and soles + insomnia. Among them, the highest confidence was obtained for feverish sensation in the chest, palms, and soles + spontaneous sweating (72.92%). In other words, yin deficiency was diagnosed in 72.92% of cases where feverish sensation in the chest, palms, and soles + spontaneous sweating were reported. The results are shown in Table 9 and Figure 4.

## 4. Discussion

Identification of the pathological nature of the disease based on syndrome differentiation is the core of treatment in TCM [20].

### 4.1. Distribution of Syndromes in CHF

The syndromes identified in this study, in descending order of frequency, were qi deficiency, blood stasis, fluid retention, yin deficiency, phlegm turbidity, yang deficiency, blood deficiency, and qi stagnation. The most frequent disease site was the heart, followed by the kidney, spleen, lung, and liver. A review of the last 10 years of literature [6] on the distribution of TCM syndromes in CHF revealed that the six most frequent syndromes in descending order were qi deficiency, blood stasis, yang deficiency, yin deficiency, fluid retention, and phlegm turbidity; the three most frequent disease sites in descending order were the heart, lung, and kidney. This is essentially consistent with the frequency of syndromes and disease sites observed in this study.

Moreover, the distribution of syndromes in patients with CHF of different cardiac function classes suggested that qi deficiency and blood stasis are always involved in the pathological process of CHF. This is related to the distribution of syndromes in coronary heart disease, the underlying cause of CHF. A questionnaire survey conducted by Liu [21] in 2013 showed that the primary syndromes were yang deficiency and blood stasis among 1504 patients with coronary heart disease with heart failure.

### 4.2. Comparison of Syndrome Distribution in Patients with CHF of Different Cardiac Function Classes

This study shows that the frequency of syndromes in patients with CHF varied with cardiac function class. Specifically, yang deficiency and fluid retention occurred at a higher frequency in classes III and IV than in classes I and II, whereas the opposite was true for phlegm turbidity. Moreover, the frequency of yang deficiency, phlegm turbidity, and fluid retention was significantly different between the different cardiac function classes at a statistically significant level. Obviously, the syndrome in the CHF patients changed with the deterioration of cardiac function. In patients with heart failure, long-term qi deficiency impairs yin and, in severe cases, yang. The insufficient generation or excessive consumption of heart-yang results in pathological changes, including heart-qi deficiency, insufficiency of heart-yin, heart-yang deficiency, heart-blood stasis, phlegm turbidity, and fluid retention. Hence, the symptoms and signs of yang deficiency are more obvious in late heart failure. Therefore, there were certain syndromes and pathogenetic characteristics at different stages of heart failure. The more severe the disease, the worse the heart-qi (yang) deficiency [22].

The disease sites were basically the same among patients of different cardiac function classes. The primary disease site was the heart, and other common disease sites included the kidney, spleen, and lung. The disease sites dynamically changed with the cardiac function class. This is consistent with the assertion of Tietao Deng that “all five viscera, not only the heart, can lead to heart failure” [23]. Specifically, dysfunction of all five viscera can affect the occurrence and development of heart failure, and the exhaustion of qi, blood, yin, and yang in the heart can also result in dysfunction of qi, blood, yin, and yang in other viscera.

### 4.3. Association Rules between High-Frequency Syndromes and Symptoms in Patients with CHF

Syndromes are the basic elements of syndrome differentiation. In recent years, many scholars have conducted in-depth research on syndrome differentiation methods and proposed a new syndrome differentiation method system using syndrome combinations for syndrome differentiation, which provides a new method to reveal the law, essence, and characteristics of syndrome differentiation, as well as the standardization of syndrome differentiation[24]. However, many scholars have found that due to the different characteristics of different diseases and different contributions of related factors, the manifestations of their syndrome are also different; therefore, a generalized diagnosis cannot meet clinical needs [25]. Therefore, in this study, association rule analysis of high-frequency syndromes and symptoms was added in order to elucidate the rules between them in patients with chronic heart failure.

Qi deficiency is one of the important pieces of evidence and a common TCM syndrome differentiation type, which generally refers to a series of manifestations caused by weak constitution or long illness. As early as in* NeiJing*, there are elaborations on “insufficiency of qi” and “qi deficiency”. For example, people with qi deficiency often feel tired and weak, having a low faint voice, lack of energy, shortness of breath, pale complexion, inappetence, head and limbs oedema, borborygmus and loose stools, dyspepsia, hidrosis and spontaneous sweating, weak pulse, pale tongue fat, or tooth marks, which are all signs of qi deficiency [26]. In patients with chronic heart failure with qi deficiency, according to the association rules between qi deficiency and symptoms, common symptoms include fatigue, mental fatigue, shortness of breath, chest tightness, dyspnoea, insomnia + spiritlessness, thready pulse + shortness of breath, and moist tongue coating + spiritlessness. Qi plays an important role in the human body. The production, distribution, and excretion of essence, blood, and body fluid depend on the promotion of qi. In the book of* Nan Jing*, he said, “qi is the root of the human”. If the promoting effect of qi is weakened, there will be underproduction and obstruction of the distribution of fluid-blood.

Blood stasis mainly refers to various factors causing stasis, such as qi stagnation, qi deficiency, phlegm retention, cold coagulation, blood heat, and trauma, leading to poor blood flow and blocked arteries. Its clinical manifestations are characterized by dull tongue, petechiae or ecchymosis, sublingual varices, cyanosis of the lips, dryness, and thin pulse [27]. In chronic heart failure, according to the association rules between blood stasis and symptoms, the common symptoms are chest tightness, palpitations, shortness of breath, and spontaneous sweating + cyanosis of lips. As blood is key to qi, it can nourish and carry qi. Normal blood flow depends on promoting and controlling functions of heart qi. Sufficient heart qi can promote normal blood flow in the veins, such that it does not overflow. The heart-qi deficiency will lead to poor blood flow and stasis. Blood stasis in the heart will lead to chest tightness and palpitations. Blood stasis in the lung will lead to cough or haemoptysis, dyspnoea, and an inability to lie down.

Fluid retention refers to the pathological products of water and liquid metabolism disorder formed in the body. Common symptoms include dizziness, chest distress, vomiting water, salivation, greasy hair, and thready and slippery pulse. Fluid retention occurs in different places, which has different effects [28]. According to the association rules between fluid retention and symptoms, the common symptoms are hard pulse + oedema, difficult expectoration + oedema, hypochondriac distension + spontaneous sweating, nausea + hypochondriac distension, hypochondriac distension + inability to lie flat, and hypochondriac distension + oedema. In patients with chronic heart failure, the initial stage is dominated by heart-qi deficiency. With further progression of the disease, heart-yang deficiency gradually appears, leading to kidney yang deficiency, loss of perspiration and gasification function, and imbalance of water and fluid metabolism. The heart-yang qi deficiency does not help the lung, which leads to concomitant heart and lung disease. Lung qi deficiency leads to the loss of water channel regulation and fluid stoppage. Heart-yang deficiency cannot warm the spleen yang, leading to heart and spleen disease at the same time. The spleen is incapacitated, unable to transport water, causing water-dampness retention, affecting the heart and lung. Therefore, the clinical manifestations include palpitations, asthma, chest distress, and cough. When the water-dampness overflows into the skin, oedema of the face and limbs occurs. Fluid retention is the inevitable result of chronic heart failure. In clinical practice, we can combine the results of association rules, establish the pattern of the syndrome, and conduct syndrome differentiation for treatment.

Yin deficiency refers to deficiency of blood or body fluid. Clinical manifestations mainly include hot flushes; red cheeks; night sweating; feverish sensation in the chest, palms, and soles; haemoptysis; blurred vision; emaciation or insomnia; dysphoria; spermatorrhea; hypersexuality; red tongue; and dry mouth [29]. In chronic heart failure, according to the association rule between yin deficiency and symptoms, the common symptoms include feverish sensation in the chest, palms, and soles + spontaneous sweating; red tongue + thready pulse; red tongue + dry mouth; and feverish sensation in the chest. Yin and yang require each other and are inseparable. According to* The Su-Wen · The Theory of Natural Gas*, “Yang qi is rooted in yin, and yin qi is rooted in yang. Yang is not born without yin, and yin does not change without yang.” Because yin and yang are mutually rooted, qi and blood depend on each other. Therefore, yin blood deficiency means that the blood does not support the heart; heart qi no longer functions, leading to heart failure.

According to the clinical manifestations related to chronic heart failure, most scholars believe that heart failure belongs to the root deficiency and enrichment of symptoms. In recent years, many doctors [30] have supplemented the pathogenesis of chronic heart failure with their own clinical experience and classified chronic heart failure according to the different clinical manifestations of patients. In clinical practice, the results of association rules can be combined with the patient's symptoms to establish the syndrome type and conduct syndrome differentiation for treatment.

In this study, the distribution of syndromes in patients with CHF of different cardiac function classes was investigated. The results reflect the distribution pattern of TCM syndromes in CHF. Moreover, the correlation between high-frequency syndromes and symptoms was explored in terms of association rules, which provide guidance for the diagnosis and treatment of CHF in clinical Chinese medicine practice. The main limitations of this study are that the number of patients in each cardiac function classes varied greatly and that CHF was complicated by other diseases in most patients, which might cause some errors in the survey results. Therefore, a large-sample, standardized clinical epidemiological study of syndromes is required.

## 5. Conclusions

In terms of correlation with NYHA cardiac function classification, the frequency of yang deficiency and fluid retention was higher and that of phlegm turbidity was lower in classes III and IV than in classes I and II. This is the basic pattern observed in the evolution of syndromes in patients with CHF as cardiac function changes.

## Figures and Tables

**Table 1:** Syndromes in patients with CHF.

Syndrome	Frequency (N)	Percentage (%)
Qi deficiency	815	79.36
Blood stasis	795	77.41
Fluid retention	305	29.70
Yin deficiency	228	22.20
Phlegm turbidity	215	20.93
Yang deficiency	186	18.11
Blood deficiency	47	4.58
Qi stagnation	15	1.46
Other	11	1.07
Cold coagulation	5	0.49
Dryness-heat	2	0.19

**Table 2:** Disease sites in patients with CHF.

Disease site	Frequency (N)	Percentage (%)
Heart	907	88.32
Kidney	324	31.55
Lung	295	28.72
Spleen	293	28.53
Liver	97	9.44
Stomach	55	5.36
Gallbladder	8	0.78

**Table 3:** Distribution of syndromes in patients with CHF of different cardiac function classes.

Syndrome	Class I cardiac function		Class II cardiac function		Class III cardiac function		Class IV cardiac function	
	Frequency (N)	Percentage (%)	Frequency (N)	Percentage (%)	Frequency (N)	Percentage (%)	Frequency (N)	Percentage (%)
Blood stasis	8	72.73	211	73.78	487	79.84	89	74.17
Qi deficiency	7	63.64	226	79.02	488	80	94	78.33
Yin deficiency	5	45.45	70	24.48	121	19.84	32	26.67
Phlegm turbidity	3	27.27	72	25.17	105	17.21	35	29.17
Fluid retention	1	9.09	27	9.44	208	34.1	69	57.50
Yang deficiency	1	9.09	19	6.64	130	21.31	36	30.00
Blood deficiency			12	4.20	35	5.74		
Qi stagnation			10	3.50	4	0.66	1	0.83
Cold coagulation			5	1.75				
Dryness-heat			2	0.70				

**Table 4:** Distribution of disease sites in patients with CHF of different cardiac function classes.

Disease site	Class I cardiac function		Class II cardiac function		Class III cardiac function		Class IV cardiac function	
	Frequency (N)	Percentage (%)	Frequency (N)	Percentage (%)	Frequency (N)	Percentage (%)	Frequency (N)	Percentage (%)
Heart	9	81.82	244	85.31	541	88.69	113	94.17
Kidney	4	36.36	70	24.48	192	31.48	58	48.33
Spleen	4	36.36	71	24.83	168	27.54	50	41.67
Lung	2	18.18	56	19.58	194	31.8	43	35.83
Liver	1	9.09	32	11.19	61	10	3	2.5
Stomach			17	5.94	32	5.25	6	5
Gallbladder			1	0.35	7	1.15	1	0.83

**Table 5:** Comparison of distribution of yin deficiency, yang deficiency, phlegm turbidity, and fluid retention between different NYHA cardiac function classes.

Syndrome	Cardiac function class	Yes	No	*χ*2	P
Yin deficiency	Class I	5 (45.5%)	6 (54.5%)	9.914	0.078
	Class II	70 (24.5%)	216 (75.5%)		
	Class III	121 (19.8%)	489 (80.2%)		
	Class IV	32 (26.7%)	88 (73.3%)		
	Class I + Class II	75(25.3%)	222 (74.7%)		
	Class III + Class IV	153(21.0%)	577(79.0%)		
Yang deficiency	Class I	1 (9.1%)	10 (90.9%)	1935.984	0.001
	Class II	19 (6.6%)	267 (93.4%)		
	Class III	130 (21.3%)	480 (78.7%)		
	Class IV	36 (30.0%)	84 (70.0%)		
	Class I + Class II	20(6.7%)	277 (93.3%)		
	Class III + Class IV	166 (22.7%)	564 (77.3%)		
Phlegm turbidity	Class I	3 (27.3%)	8 (72.7%)	18.097	0.003
	Class II	72 (25.2%)	214 (74.8%)		
	Class III	105 (17.2%)	505 (82.8%)		
	Class IV	35 (29.2%)	85 (70.8%)		
	Class I + Class II	75 (25.3%)	222 (74.7%)		
	Class III + Class IV	140 (19.2%)	590 (80.8%)		
Fluid retention	Class I	1 (9.1%)	10 (90.9%)	190.765	0.001
	Class II	27 (9.4%)	259 (90.6%)		
	Class III	208 (34.1%)	402 (65.9%)		
	Class IV	69 (57.5%)	51 (42.5%)		
	Class I+ Class II	28 (9.4%)	269 (90.6%)		
	Class III + Class IV	277 (37.9%)	453 (62.1%)		

**Note: **Yang deficiency: class II vs. class III, P < 0.05; class II vs. class IV, P < 0.05; class III vs. class IV, P < 0.05; classes I + II vs. classes III +IV, P < 0.05.

Phlegm turbidity: class II vs. class III, P < 0.05; class III vs. class IV, P < 0.05; classes I + II vs. classes III +IV, P < 0.05.

Fluid retention: class I vs. class IV, P < 0.05; class II vs. class III, P < 0.05; class II vs. class IV, P < 0.05; class III vs. class IV, P < 0.05; classes I + II vs. classes III +IV, P < 0.05.

**Table 6:** Association rules between qi deficiency and symptoms.

Consequent	Antecedent	Support (%)	Confidence (%)
Qi deficiency = T	Insomnia = T and Spiritlessness = T	58.10	87.43
Qi deficiency = T	Thready pulse = T and Shortness of breath = T	50.00	86.98
Qi deficiency = T	Moist tongue coating = T and Spiritlessness = T	51.43	86.73
Qi deficiency = T	Insomnia = T and Shortness of breath = T	60.32	86.58
Qi deficiency = T	Soreness and weakness of waist and knees = T and Spiritlessness = T	62.06	86.45
Qi deficiency = T	Insomnia = T and Weak breathing with no desire to speak = T	50.00	86.35
Qi deficiency = T	Insomnia = T and Weakness = T	62.54	86.29
Qi deficiency = T	Thready pulse = T and Weakness = T	51.43	86.11
Qi deficiency = T	Wiry pulse = T and Spiritlessness = T	52.22	86.02
Qi deficiency = T	Thready pulse = T	51.75	85.89
Qi deficiency = T	Insomnia = T and Palpitation = T	50.48	85.85
Qi deficiency = T	Insomnia = T	63.49	85.75
Qi deficiency = T	Insomnia = T and Chest tightness = T	58.73	85.68
Qi deficiency = T	Spiritlessness = T and Shortness of breath = T	84.76	85.58
Qi deficiency = T	Spiritlessness = T	88.89	85.54

T= true, indicating the presence of the syndrome or symptom.

**Table 7:** Association rules between blood stasis and symptoms.

Consequent	Antecedent	Support (%)	Confidence (%)
Blood stasis = T	Spontaneous sweating = T and Cyanosis of lips = T	31.43	84.85
Blood stasis = T	Night sweats = T and Cyanosis of lips = T	35.56	83.04
Blood stasis = T	Thready pulse = T and Cyanosis of lips = T	33.65	82.55
Blood stasis = T	Oedema = T and Cyanosis of lips = T	40.32	82.28
Blood stasis = T	Wiry pulse = T and Cyanosis of lips = T	40.95	82.17
Blood stasis = T	Chest pain = T and Weakness = T	31.11	82.14
Blood stasis = T	Chest pain = T	32.22	81.77
Blood stasis = T	Cyanosis of nails = T and Spiritlessness = T	30.48	81.25
Blood stasis = T	Abdominal distension = T and Cyanosis of lips = T	31.11	81.12
Blood stasis = T	Cyanosis of nails = T and Chest tightness = T	31.90	81.09
Blood stasis = T	Chest pain = T and Chest tightness = T	30.79	80.93
Blood stasis = T	Cough = T and Cyanosis of lips = T	33.17	80.86
Blood stasis = T	Cyanosis of nails = T and Shortness of breath = T	31.43	80.81
Blood stasis = T	Insomnia = T and Cyanosis of lips = T	44.13	80.58
Blood stasis = T	Moist tongue coating = T and Cyanosis of lips = T	36.67	80.52

T= true, indicating the presence of the syndrome or symptom.

**Table 8:** Association rules between fluid retention and symptoms.

Consequent	Antecedent	Support (%)	Confidence (%)
Fluid retention = T	Hard pulse = T and Oedema = T	11.43	75.00
Fluid retention = T	Difficult expectoration = T and Oedema = T	10.32	72.31
Fluid retention = T	Hypochondriac distension = T and Spontaneous sweating = T	12.70	68.75
Fluid retention = T	Nausea = T and Hypochondriac distension = T	10.63	68.66
Fluid retention = T	Hypochondriac distension = T and Inability to lie flat = T	10.48	68.18
Fluid retention = T	Hypochondriac distension = T and Oedema = T	16.35	67.96
Fluid retention = T	Thirst without desire to drink = T and Thready pulse = T	10.79	67.65
Fluid retention = T	Oliguria = T and Thready pulse = T	10.79	67.65
Fluid retention = T	Nausea = T and Oedema = T	14.60	66.30
Fluid retention = T	Thirst without desire to drink = T and Insomnia = T	10.79	66.18
Fluid retention = T	Abdominal distension = T and Oedema = T	27.14	66.08
Fluid retention = T	Hypochondriac distension = T and Night sweats = T	13.49	65.88
Fluid retention = T	Thirst without desire to drink = T and Cyanosis of lips = T	11.11	65.71
Fluid retention = T	Fat tongue = T and Inability to lie flat = T	10.16	65.63
Fluid retention = T	Jugular venous distention = T and Oedema = T	12.70	65.00

T= true, indicating the presence of the syndrome or symptom.

**Table 9:** Association rules between yin deficiency and symptoms.

Consequent	Antecedent	Support (%)	Confidence (%)
Yin deficiency = T	Feverish sensation in the chest, palms and soles = T and Spontaneous sweating = T	15.24	72.92
Yin deficiency = T	Red tongue = T and Thready pulse = T	11.75	70.27
Yin deficiency = T	Red tongue = T and Dry mouth = T	10.79	69.12
Yin deficiency = T	Feverish sensation in the chest, palms, and soles = T and Night sweats = T	17.94	67.26
Yin deficiency = T	Feverish sensation in the chest, palms, and soles = T and Dysphoria = T	13.65	66.28
Yin deficiency = T	Fissured tongue = T and Dry mouth = T	10.79	66.18
Yin deficiency = T	Red tongue = T and Wiry pulse = T	11.59	65.75
Yin deficiency = T	Feverish sensation in the chest, palms, and soles = T and Dizziness = T	19.21	63.64
Yin deficiency = T	Feverish sensation in the chest, palms, and soles = T and Thready pulse = T	15.24	63.54
Yin deficiency = T	Feverish sensation in the chest, palms, and soles = T and Dry mouth = T	20.00	62.70
Yin deficiency = T	Feverish sensation in the chest, palms, and soles = T and Insomnia = T	18.10	62.28
Yin deficiency = T	Dysphoria = T and Thready pulse = T	14.29	62.22
Yin deficiency = T	Feverish sensation in the chest, palms, and soles = T and Abdominal distension = T	13.33	60.71
Yin deficiency = T	Feverish sensation in the chest, palms, and soles = T and Palpitation = T	21.59	60.29
Yin deficiency = T	Feverish sensation in the chest, palms, and soles = T and Giddiness = T	12.70	60.00

T= true, indicating the presence of the syndrome or symptom.

## Data Availability

The data used to support the findings of this study have not been made available to protect the subjects' privacy.
